# Salmonellosis outbreaks linked to eggs at 2 gimbap restaurants in Korea

**DOI:** 10.4178/epih.e2024036

**Published:** 2024-03-07

**Authors:** Jun Suk Eun, Joohyun Han, Ji-Hyun Lim, Eunkyung Shin, Junyoung Kim, Da-Jung Ko, Jaeil Yoo, Sungil Kim, Jin Sook Kim, Jung Sub Park, Ji-Hyuk Park

**Affiliations:** 1 Disease Management Division, Deogyang-gu Public Health Center, Goyang, Korea; 2 Department of Epidemiology and Health Promotion, Graduate School of Public Health, Yonsei University, Seoul, Korea; 3 Division of Bacterial Diseases, Bureau of Infectious Disease Diagnosis Control, Korea Disease Control and Prevention Agency, Cheongju, Korea; 4 Microbiological Inspection Team, Gyeonggi Province Institute of Health and Environment in North Branch, Uijeongbu, Korea; 5 Foodborne Disease Prevention & Surveillance Division, Food and Consumer Safety Bureau, Ministry of Food and Drug Safety, Cheongju, Korea; 6 Department of Preventive Medicine, Dongguk University College of Medicine, Gyeongju, Korea

**Keywords:** *Salmonella* Enteritidis, Foodborne diseases, Outbreaks, Molecular typing

## Abstract

**OBJECTIVES:**

Salmonellosis outbreaks occurred at 2 restaurants 2 days apart, and an epidemiological investigation was conducted to determine whether the outbreaks were connected.

**METHODS:**

Case studies were conducted for both outbreaks. Stool samples were collected from individuals, and food samples were collected from the restaurants. Pulsed-field gel electrophoresis (PFGE) and whole-genome sequencing analyses were performed on outbreak-related *Salmonella enterica* serovar Enteritidis (*Salmonella Enteritidis*) isolates. Traceback investigations were also conducted for the ingredients from gimbap restaurants A and B.

**RESULTS:**

In total, 106 people from gimbap restaurant A and 5 from gimbap restaurant B met the case definition. *Salmonella* Enteritidis was detected in samples from 2 food handlers, 22 patients, and 1 food (iceberg lettuce) at gimbap restaurant A and from 1 patient at gimbap restaurant B. According to PFGE, all isolates were identified as SEGX01.089. The molecular typing of all isolates showed the same pattern, and the genetic distance was close according to phylogenetic analysis. Eggs were the only food ingredient that was supplied to both gimbap restaurants.

**CONCLUSIONS:**

The outbreaks were caused by *Salmonella* Enteritidis, and the source of infections was suspected to be contaminated eggs. To prevent foodborne outbreaks of *Salmonella*, restaurants should heat eggs sufficiently, and egg farms need to establish management systems that prevent *Salmonella* infections.

## INTRODUCTION

Foodborne diseases are caused by the contamination of food or water with pathogenic microorganisms [1]. In Korea, an outbreak of a foodborne disease is defined as the occurrence of symptoms such as diarrhea and vomiting in 2 or more individuals who have consumed the same food or water [2]. In 2022, the most common cause of bacterial gastroenteritis was norovirus, accounting for 23.5% of cases, followed by *Campylobacter* spp. (16.0%) and *Salmonella* spp. (15.5%) [3].

Non-typhoidal *Salmonella* (NTS) is a significant cause of bacterial diarrhea [4] and is responsible for an estimated 95 million cases of enterocolitis globally, along with approximately 50,771 deaths each year [5]. In Korea, *Salmonella* spp. constituted the second most common cause of foodborne outbreaks between 2021 and 2022, and the third most common in 2020 [2,6]. Restaurants were the source of the majority of *Salmonella* outbreaks, accounting for 61.8% of cases during the period from 2018 to 2022 [7].

NTS infections are generally characterized by acute-onset inflammatory diarrhea, abdominal pain, nausea, and vomiting, with an incubation period ranging from 6 hours to 72 hours following ingestion [8]. Major reservoirs of NTS include animals such as poultry, reptiles, and amphibians [9]. Transmission of NTS generally occurs through the consumption of contaminated food or beverages, including eggs, dairy products, or water [10].

The Enteric Pathogens Active Surveillance Network in Korea actively monitors *Salmonella* spp. and other foodborne pathogens [11]. In 2019, there were 653 isolates of *Salmonella* spp. identified, followed by 610 isolates in 2020, and 609 isolates in 2021. The predominant serotypes identified were *Salmonella enterica* serovar Enteritidis (*Salmonella* Enteritidis), with 379 isolates (20.2%), *S. enterica* serovar Typhimurium, with 357 isolates (19.1%), and *S. enterica* serovar I 4,[5],12:i:-, with 301 isolates (16.1%) [12].

In late August 2021, a foodborne disease outbreak was reported at restaurant A in Goyang City, and 2 days later, another outbreak occurred at restaurant B in the same city. We conducted epidemiological investigations to identify the causes of these outbreaks and to determine whether they were related.

## MATERIALS AND METHODS

### Outbreak setting and case definition

On August 25, 2021, 2 separate groups (8 office workers and 9 hospital staff) reported suspected foodborne disease outbreaks to the same public health center. Both groups had consumed food from restaurant A on August 23. On August 27, the public health center received 2 additional reports of suspected foodborne disease outbreaks from 2 other groups: a pair of acquaintances and a family of 3. These groups had consumed food from restaurant B on the afternoon of August 23. Following the report of a fatality on August 25, a joint epidemiological investigation was launched to assess the significance of the death. This investigation involved the public health center, the Gyeonggi Provincial Government, the Seoul Regional Food and Drug Administration, and the Ministry of Food and Drug Safety (MFDS). The cases were categorized as either probable or confirmed. Probable cases were defined as individuals experiencing illness with diarrhea (defined as ≥ 3 loose stools within any 24-hour period between August 23 and 26). Confirmed cases were those with a positive result for NTS among patrons or food handlers from restaurants A and B.

### Epidemiological investigation and site inspection

Case studies were conducted due to the challenges of establishing appropriate controls. We modified standardized questionnaires from the Korea Disease Control and Prevention Agency (KDCA) to gather demographic characteristics, clinical data, and histories of food and water consumption at restaurants A and B [13]. Individuals who provided stool samples were interviewed in person, while those who did not were interviewed by telephone. On August 25 and August 27, we inspected the kitchens of restaurants A and B, respectively. During these inspections, we interviewed food handlers to examine cooking procedures and collected samples of food ingredients.

### Collection of specimens and bacterial isolation

Food workers were required to provide mandatory samples, and patients exhibiting symptoms were asked to submit stool samples. Environmental specimens, which included cooking utensils (18 from restaurant A and 24 from restaurant B), food ingredients and processed foods (14 and 13, respectively), and leftover side dishes (6 from each restaurant), were collected on August 25 at restaurant A and on August 27 at restaurant B. These samples were tested for 10 bacterial species and 5 viral species known to cause gastrointestinal infections. Laboratory analyses were conducted at the Institute of Health and Environment using methods in line with the KDCA’s Foodborne Disease Laboratory Diagnosis Guidelines [14]. Samples of cooking and drinking water were also taken. The cooking water was tested for fecal coliforms, total colony counts, total coliforms, and free residual chlorine. The drinking water was tested for *Escherichia* coli and *Salmonella* spp. Egg samples from restaurant A, collected on August 25, and from restaurant B, collected on August 26 (both sourced from the same farm), were tested for *Salmonella* spp. at the Veterinary Service Laboratory. To detect species-specific genes of bacterial foodborne pathogens, polymerase chain reaction assays were carried out following the KDCA’s guidelines [14].

### Genetic characterization

Additional genetic characterization and molecular epidemiological analysis were conducted by the KDCA. All isolates underwent antimicrobial susceptibility testing (AST) using the customized Sensititre panel KRCDC2F (Trek Diagnostics Systems, Cleveland, OH, USA) in accordance with the Clinical and Laboratory Standards Institute guidelines. Furthermore, pulsed-field gel electrophoresis (PFGE) was carried out using the XbaI restriction enzyme, following the PulseNet International protocol (https://pulsenetinternational.org/protocols/pfge/). The genetic relatedness between PFGE patterns was determined using BioNumerics v7.6 (Applied Maths, Sint-Martens-Latem, Belgium). Whole genome sequencing (WGS) was utilized for detailed molecular epidemiological analysis. Sequencing was executed on the MiSeq platform as per the Illumina protocol. WGS data were analyzed to identify sequence type (MLST 2.0), resistance genes (ResFinder 4.1), and *Salmonella* pathogenicity islands (SPI; SPIFinder 2.0) using the Center for Genomic Epidemiology (CGE) web-based bioinformatics platform. Phylogenetic analysis, based on the identified single nucleotide polymorphism (SNP) variants, was performed using CSIPhylogeny from CGE. The core genome multilocus sequence typing (cgMLST) analysis and minimum spanning tree (MST) were generated based on a scheme of 3,002 target loci of *S. enterica* using the SeqSphere program.

### Traceback investigation

The distribution of products was monitored by checking the purchase dates and locations of ingredients from retailers and wholesalers utilized by restaurants A and B. The public health center conducted investigations into the food ingredients and their suppliers, while the MFDS examined the nationwide distribution channels.

### Statistical analysis

We conducted an epidemiological analysis to compare the outbreak cases at restaurants A and B. The Mann-Whitney U test was utilized to evaluate differences in the incubation period by sex, place of exposure, and the presence of vomiting symptoms. Age-related differences and the frequency of diarrhea were examined through categorization and analyzed using the Kruskal-Wallis test. Data analysis was performed using Microsoft Excel 2019 for Windows (Microsoft, Redmond, WA, USA), and statistical analyses were carried out with R version 4.2.2 (R Foundation for Statistical Computing, Vienna, Austria). A p-value < 0.05 was considered statistically significant.

### Ethics statement

Institutional review board approval was not required for this study as it pertained to a public health investigation aimed at controlling infectious disease outbreaks.

## RESULTS

**Figure 1.**
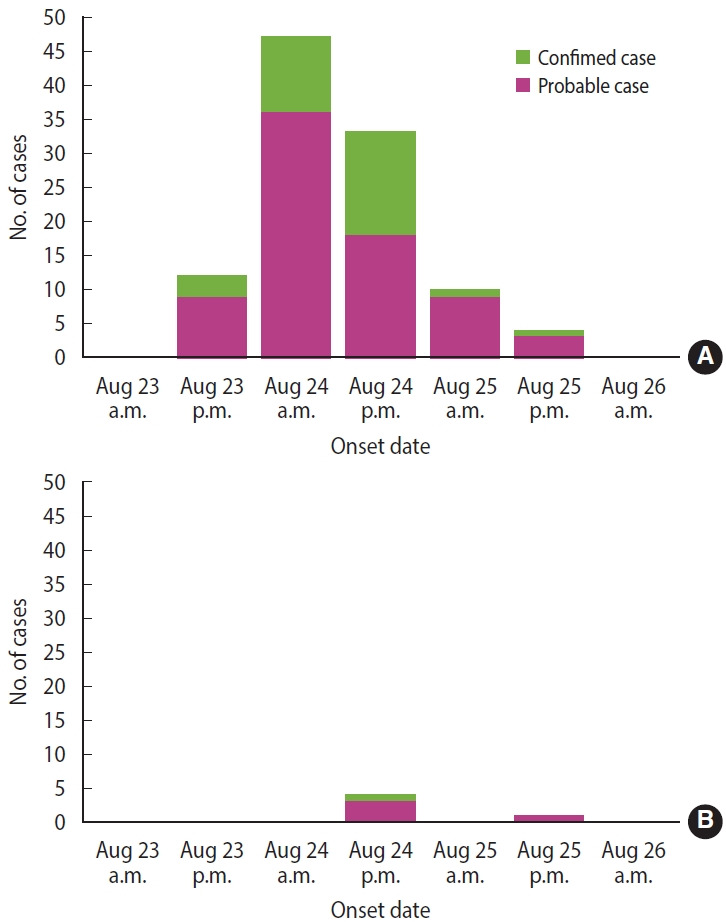
Epidemic curve of the outbreaks in restaurants A (A) and B (B; symptomatic cases).

At restaurant A, 106 people (103 out of 118 patients and 3 out of 4 food handlers) met the case definition, and 24 cases were confirmed. The most common symptom was diarrhea (98.1%), followed by abdominal pain (84.9%), fever (84.0%), and chills (65.1%) (Table 1). The outbreak started in the early afternoon of August 23 and peaked on the morning of the next day (Figure 1A). At restaurant B, 5 patients met the case definition, and 1 case was confirmed. The main reported symptoms were diarrhea (100%), abdominal pain (100%), and fever (80.0%) (Table 1). The outbreak began in the early afternoon of August 24 and peaked in the afternoon of the same day (Figure 1B). There were no asymptomatic cases at either restaurant. The incubation period was calculated as the time elapsed from the time of ingestion to the onset of symptoms. The median incubation periods at restaurants A and B were 19.5 hours (range, 3.0-48.0) and 25.0 hours (range, 20.5-46.0), respectively (Supplementary Material 1). Restaurant A had a shorter median incubation period than restaurant B, but without statistical significance (p= 0.083). The incubation periods were significantly shorter in patients with diarrhea (p= 0.039) and vomiting (p= 0.003) (Table 2).

The people tested from restaurant A consumed food between August 22 and August 24. The proportion of gimbap consumption was the highest, at 83.0% (88/106). After adding people who consumed the set menu, including gimbap, the proportion of gimbap consumption increased to 84.9% (90/106; 5 duplicated cases). All food and water items were consumed before the onset of symptoms. The same kind of food that was consumed on different dates was combined into 1 food item (Table 3). Food from restaurant B was served between 4 p.m. and 5 p.m. on August 23, and all 5 cases consumed “well-being gimbap” (Supplementary Material 2).

Of the stool samples collected from patients at restaurant A, 22 out of 46 (47.8%) were positive for *Salmonella* Enteritidis, as were 2 out of 4 (50.0%) samples from food handlers. At restaurant B, stool samples were available from 3 patients and 6 food handlers; 1 patient sample (33.3%) tested positive for *Salmonella* Enteritidis. Additionally, *Salmonella* Enteritidis was isolated from iceberg lettuce at restaurant A on August 25. All other food samples from both restaurants tested negative. Egg and water samples taken from restaurant A on August 25 and from restaurant B on August 26 were also negative for *Salmonella* Enteritidis. Samples of cooking water and drinking water from both restaurants were negative for *Salmonella* spp. and *E. coli*.

**Figure 2.**
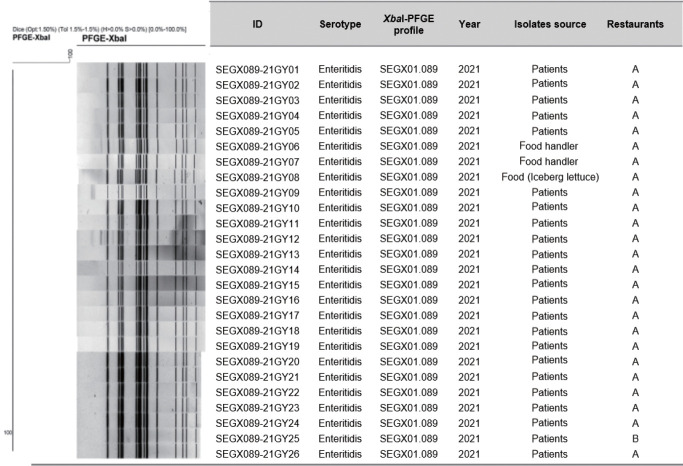
*Xba*I-PFGE pattern of isolated *Salmonella enterica* serovar Enteritidis from 2 outbreaks. A dendrogram was built using Dice/UPGMA clustering with 1.5% position tolerance. Sample ID, serotype, PFGE profile, year of isolation, isolate source and restaurants are displayed next to the dendrogram. PFGE, pulsed-field gel electrophoresis.

**Figure 3.**
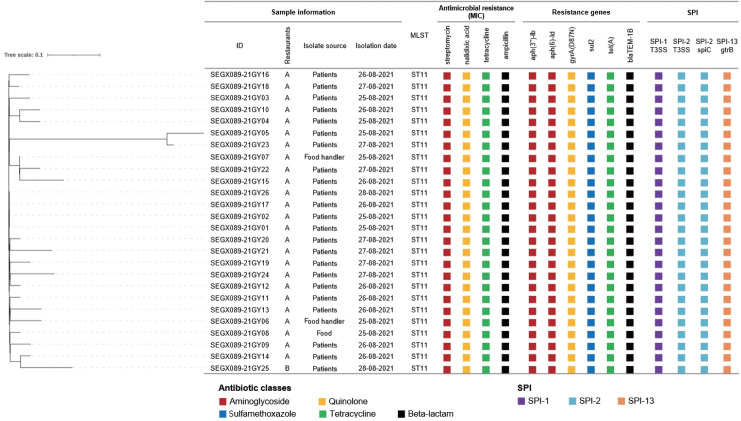
Phylogenetic tree based on SNPs and comparison of WGS analysis results and antibiotic patterns of isolated *Salmonella enterica* serovar Enteritidis from 2 outbreaks. The phylogenetic tree was constructed using the FastTree method and filtering SNPs using a z-score cut-off value of 1.96. Next to the phylogenetic tree, sample data, sequence type, detected antibiotic resistance information, and SPI are listed. The color labels of resistance parts indicate differences in antibiotic classes. SNPs, single nucleotide polymorphism; WGS, whole genome sequencing; MLST, multilocus sequence typing; SPI, *Salmonella* pathogenicity islands.

**Figure 4.**
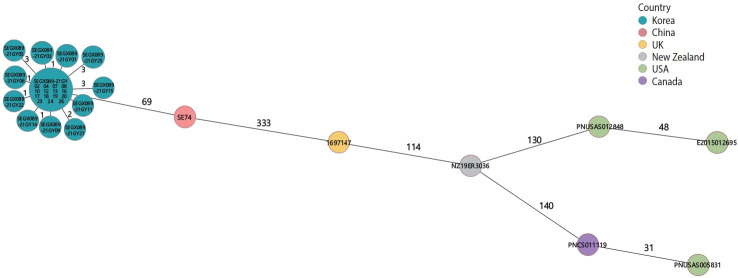
Minimum spanning tree (MST) of isolated *Salmonella enterica* serovar Enteritidis (*Salmonella* Enteritidis) from 2 outbreaks based on cgMLST for genome comparison with overseas *Salmonella* Enteritidis isolates. An MST was constructed based on the cgMLST scheme (3,002 loci) of *Salmonella* Enteritidis (P125109), and the value of connecting lines indicates the number of allelic differences between isolates. The size of the node is proportional to the number of genomes for each cgMLST genotype. The node color indicates the country of isolates from diarrhea patients. cgMLST, core genome multilocus sequence typing.

AST showed resistance to streptomycin, nalidixic acid, tetracycline, and ampicillin in all isolates. The PFGE patterns of the 26 isolates were classified as SEGX01.089 (Figure 2). The following antibiotic-resistance genes were detected: the *aph(3’’)‑Ib, aph(6)‑IId, sul2, tet*(A), *bla*_TEM-1B_ genes and the gyrA(D87N) point mutation in all isolates; all isolates had sequence type ST11 and possessed SPI sets (SPI-1 [T3SS], SPI-2 [T3SS, *spiC*], and SPI-13 [*gtrB*]) (Figure 3). A phylogenetic tree was constructed based on SNPs, forming only 1 cluster and showing 0-21 pairwise genetic distances (average, 5.8; median, 4). Additionally, MST visualized by cgMLST typing was defined as a closely related cluster with a maximum of 3 allele distances between 2 outbreak isolates. A separate cluster was formed with at least 69 allele differences from isolates from other countries (Figure 4).

The investigation into the supply chains for the food ingredients at restaurants A and B showed that while the retailers and wholesalers were different, both establishments received their eggs from farm Z in Pocheon City (Supplementary Material 3). Additionally, firm V delivered eggs to both restaurants A and B on the same dates: August 20, August 23, and August 24. The food handlers indicated that the eggs supplied on August 20 were likely used up by August 23.

## DISCUSSION

In this study, we identified a connection between 2 restaurants where foodborne disease outbreaks occurred through epidemiological investigations and laboratory data. The outbreaks at restaurants A and B were both caused by *Salmonella* Enteritidis. The median incubation periods were 19.5 hours for restaurant A and 25.0 hours for restaurant B, aligning with the known incubation range for salmonellosis [8]. Additionally, the same strain of the bacterium (SEGX01.089) was found in case samples from both restaurants and in iceberg lettuce from restaurant A. Epidemiological and traceback investigations pointed to eggs as the likely source of the outbreaks.

Other potential transmission routes for the pathogen could have been through food handlers [15] or contaminated water [16]. However, there was no connection between the food handlers at the 2 restaurants, and it was improbable that water was the infection source. This is because many customers ordered takeout food without water, and the 2 restaurants had different water suppliers. Among the food ingredients, only eggs were supplied to both establishments, and the eggs used during the outbreak were traced back to the same farm. Eggs are a well-known source of *Salmonella* infection [17-19]. In Korea, from 2017 to 2021, eggs and egg products were responsible for 76.9% of the food poisoning cases attributed to *Salmonella* [20].

*Salmonella* spp. are primarily found on eggshells and can also be present in the contents of contaminated eggs [21,22]. The preparation of *jidan* requires cracking and mixing dozens of eggs in a large container. If any of the eggs are contaminated, the entire batch can become tainted. At restaurant A, it was highly probable that *Salmonella* Enteritidis survived in the eggs due to insufficient heating time (1-2 seconds). Studies have shown that it takes 1.2 seconds to inactivate 1 log of *Salmonella* cells at 71°C [23]. Moreover, there was a risk of bacterial proliferation since the *jidan* was prepared a day before it was served. Conversely, at restaurant B, despite the longer heating duration (over 10 seconds), the *jidan* was thicker than that at restaurant A, which may have resulted in an undercooked center.

*Salmonella* Enteritidis was also detected in trimmed iceberg lettuce prepared for making “well-being gimbap” at restaurant A. Green vegetables can be a source of *Salmonella* infection [24]. However, only 15.1% of the cases at restaurant A involved “well-being gimbap” that included iceberg lettuce (Supplementary Material 2). Furthermore, the supply channels for iceberg lettuce used in restaurants A and B were different. Therefore, we hypothesize that the contaminated iceberg lettuce sample may have been tainted by contaminated eggs.

The incubation periods were significantly shorter in patients who experienced frequent diarrhea and vomiting. Additionally, higher doses of intake were correlated with both a reduced incubation period and an increase in symptom severity [25,26]. Although not statistically significant, the shorter median incubation period observed in patrons of restaurant A compared to restaurant B may be attributable to a higher consumption of *Salmonella* Enteritidis.

There were no reported outbreaks at 11 other restaurants that received eggs from firm V, which could be due to inadequate cooking at restaurants A and B. Egg samples collected on August 25 and August 26 tested negative for *Salmonella* Enteritidis. However, these samples were taken after the outbreaks had occurred, and *Salmonella* Enteritidis is more likely to be detected in eggs when chicken immunity is compromised, such as during periods of restricted feeding [27].

Early typing was explored through phenotypic analysis, such as antibiotic resistance and serotype determination. Until recently, PFGE has been the gold standard for disease investigation [28]. WGS analysis has emerged as a new molecular typing method and is now utilized in numerous laboratories [29]. The 26 isolates— comprising 23 from patients, 2 from food handlers, and 1 from iceberg lettuce—collected from the 2 restaurants were found to be closely related through molecular epidemiological experiments. Since the isolates shared the same antibiotic resistance pattern, they were presumed to be similar pathogens [29]. In the PFGE analysis, the isolates were classified under the XbaI pattern SEGX 01.089 and demonstrated 100% genetic homology. Furthermore, NGS results indicated that the sequence type, SPI set composition, and resistance genes were identical across all isolates. A pairwise comparison revealed fewer than 21 SNPs, confirming that they were the same causative bacteria [30]. Epidemiologically, all isolates belonged to a single cluster. In the MST based on cgMLST, allelic differences between nodes were represented as connecting lines, with a high genetic correlation of 0-3 observed. There was a significant genetic divergence when compared to diarrheal disease isolates from different countries. In our study, the concordance of antibiotic resistance patterns, PFGE, and NGS profiles strongly indicated a genetic link between the isolates from the 2 restaurants. These high-quality molecular epidemiological data provide robust support for epidemiological investigations.

This investigation had several limitations that need to be considered. First, we were unable to include a control group due to difficulties associated with recruiting appropriate controls; this limitation affected the case series, and we had to consider various potential transmission routes. Second, *Salmonella* Enteritidis was detected in only one stool sample from restaurant B, which can be attributed to the small number of cases and the delay in sampling. Third, we could not test egg and environmental samples from farm Z for *Salmonella* spp. We strongly recommend adopting a One Health approach to salmonellosis outbreaks to enable comprehensive investigations of farms when epidemiologically necessary.

In conclusion, epidemiological studies and molecular epidemiological analyses have traced the source of infection to contaminated eggs. To prevent outbreaks of salmonellosis, it is imperative that restaurants handle eggs with strict hygiene and cook them thoroughly. Furthermore, systematic management of breeding environments and improved surveillance for salmonellosis at egg production facilities are essential.

## Figures and Tables

**Table 1.** Demographic and clinical characteristics of cases at restaurants A and B

Characteristics	Restaurant A (n=106)	Restaurant B (n=5)
Sex		
Male	44 (41.5)	1 (20.0)
Female	62 (58.5)	4 (80.0)
Age (yr)		
≤9	9 (8.5)	0 (0.0)
10-19	9 (8.5)	2 (40.0)
20-29	21 (19.8)	0 (0.0)
30-39	28 (26.4)	1 (20.0)
40-49	14 (13.2)	1 (20.0)
50-59	18 (17.0)	0 (0.0)
≥60	7 (6.6)	1 (20.0)
Symptoms		
Diarrhea	104 (98.1)	5 (100)
Nausea	55 (51.9)	2 (40.0)
Vomiting	35 (33.0)	0 (0.0)
Abdominal pain	90 (84.9)	5 (100)
Fever	89 (84.0)	4 (80.0)
Chills	69 (65.1)	2 (40.0)

**Table 2.** Analysis of incubation period by general characteristics

Characteristics	n (%)	Median incubation period (hr)	Interquartile range (hr)	p-value
Sex				0.342^1^
Male	45 (40.5)	18.8	14.0-24.5	
Female	66 (59.5)	21.0	14.0-26.0	
Age (yr)				0.173^2^
≤9	9 (8.1)	21.0	17.0-31.0	
10-19	11 (9.9)	23.0	19.8-29.8	
20-29	21 (18.9)	15.0	12.0-21.3	
30-39	29 (26.1)	20.5	16.0-26.0	
40-49	15 (13.5)	24.0	10.8-27.3	
50-59	18 (16.2)	18.3	14.3-24.4	
≥60	8 (7.2)	14.5	8.4-21.3	
Location				0.083^1^
Restaurant A	106 (95.5)	19.5	14.0-25.0	
Restaurant B	5 (4.5)	25.0	21.0-29.5	
Diarrhea				0.039^2^
None	2 (1.8)			
1-9	48 (43.2)	23.5	15.0-29.6	
10-19	29 (26.1)	19.0	14.0-22.0	
≥20	32 (28.8)	17.3	13.8-21.8	
Vomiting				0.003^1^
None	76 (68.5)	21.2	15.0-28.4	
≥1	35 (31.5)	17.0	12.0-20.9	

1 Mann-Whitney U test was applied.

2 Kruskal-Wallis test was applied.

**Table 3.** Food and water items consumed at restaurants A and B

Food and water	Restaurant A (n=106)	Restaurant B (n=5)
Food items		
Gimbap	88 (83.0)	5 (100)
Set menu	26 (24.5)	1 (20.0)
Including gimbap	7 (6.6)	1 (20.0)
Excluding gimbap	19 (17.9)	0 (0.0)
Soup	0 (0.0)	0 (0.0)
Stew	2 (1.9)	0 (0.0)
Flour-based food	30 (28.3)	0 (0.0)
Rice-based food	21 (19.8)	0 (0.0)
Side dish	47 (44.3)	2 (40.0)
Water items		
Purified water	10 (9.4)	1 (20.0)

## Supplementary materials

Supplementary Material 1. Raw data about incubation periods

Supplementary Material 2. Types of gimbap consumed by restaurants A and B cases

Supplementary Material 3. Food ingredients and their retailers and wholesalers at the two restaurants
