# Supplementary material for: Salmonellosis outbreaks linked to eggs at 2 gimbap restaurants in Korea
Source: Epidemiol Health. 2024 Mar 7;46:e2024036. doi: 10.4178/epih.e2024036 (PMC11369563; doi:10.4178/epih.e2024036)
Supplement: Supplementary Material 2. — Types of gimbap consumed by restaurants A and B cases [file epih-46-e2024036-Supplementary-2.docx]

Supplementary Material 2. Types of gimbap consumed by restaurants A and B cases

| Type of gimbap | Included | | Restaurant A (n=106) | Restaurant B (n=5) |
| --- | --- | --- | --- | --- |
|  | Jidan | Iceberg leuttuce |  |  |
| Regular gimbap | Yes | - | 31 (29.2) | 1 (20.0) |
| Vegetable gimbap | Yes | - | 6 (5.7) | 0 (0.0) |
| Cheese gimbap | Yes | - | 7 (6.6) | 0 (0.0) |
| Kimchi gimbap | Yes | - | 2 (1.9) | 0 (0.0) |
| Chilli gimbap | Yes | - | 1 (0.9) | 0 (0.0) |
| Tuna gimbap | Yes | - | 28 (26.4) | 0 (0.0) |
| Beef gimbap | Yes | - | 5 (4.7) | 0 (0.0) |
| Well-being gimbap | Yes | Yes | 16 (15.1) | 5 (100.0) |
| Pork cutlet gimbap | Yes | - | 2 (1.9) | 0 (0.0) |
| Pork belly gimbap | Yes | - | 15 (14.2) | 0 (0.0) |
| Beef brisket gimbap | Yes | - | 4 (3.8) | 0 (0.0) |
| Buckwheat-egg gimbap | Yes | - | 13 (12.3) | 0 (0.0) |
