# Supplementary material for: Salmonellosis outbreaks linked to eggs at 2 gimbap restaurants in Korea
Source: Epidemiol Health. 2024 Mar 7;46:e2024036. doi: 10.4178/epih.e2024036 (PMC11369563; doi:10.4178/epih.e2024036)
Supplement: Supplementary Material 3. — Food ingredients and their retailers and wholesalers at the two restaurants [file epih-46-e2024036-Supplementary-3.docx]

Supplementary Material 3. Food ingredients and their retailers and wholesalers at the two restaurants

| Retailers | Wholesalers | Food ingredients | Handling period |
| --- | --- | --- | --- |
| Restaurant A |  |  |  |
| Firm V (Eunpyeong-gu) | Firm W (Eunpyeong-gu) | Egg (Farm Z) | Aug. 20–24 |
| Discount store D | Distribution H (Gangseo-gu) | Iceberg lettuce (USA) | Aug. 21–23 |
| Fruit shop E | Distribution J (Gangseo-gu) | Iceberg lettuce (domestic) | Unknown |
|  | Agriculture J (Gangseo-gu) | Crispy chilli, cheongyang pepper, etc. | Unknown |
| Food F | Distribution K (Gangseo-gu) | Perilla leaf, romaine green lettuce | Unknown |
|  | Distribution L (Gangseo-gu) | Carrot (China) | Unknown |
|  | General trading company M | Cheese (domestic, Seoul Milk Cheddar) | Aug. 23 |
| Restaurant B |  |  |  |
| Firm V (Eunpyeong-gu) | Firm W (Eunpyeong-gu) | Egg (Farm Z) | Aug. 20–24 |
| Food mart G | Agriculture N | Iceberg lettuce, pimento, pepper, paprika | Aug. 16–23 |
|  | Distribution O (Gangseo-gu) | Leaf lettuce, perilla leaf, carrot | Aug. 16–23 |
|  | Agriculture P | Leaf lettuce, perilla leaf | Aug. 16–23 |
|  | Distribution Q (Gangseo-gu) | Perilla leaf | Aug. 16–23 |
|  | Agriculture R | Carrot (imported and domestic) | Aug. 16–23 |
|  | Agriculture S | Carrot | Aug. 16–23 |
|  | Food T (Gangseo-gu) | Burdock root (China) | Aug. 16–23 |
